# The Influence of Genetic Variations in the *CD86* Gene on the Outcome after Allogeneic Hematopoietic Stem Cell Transplantation

**DOI:** 10.1155/2018/3826989

**Published:** 2018-02-07

**Authors:** Lidia Karabon, Miroslaw Markiewicz, Karolina Chrobot, Monika Dzierzak-Mietla, Edyta Pawlak-Adamska, Anna Partyka, Anna Koclega, Slawomira Kyrcz-Krzemien, Irena Frydecka

**Affiliations:** ^1^Department of Experimental Therapy, L. Hirszfeld Institute of Immunology & Experimental Therapy, Polish Academy of Sciences, R Weigl 12, 53-114 Wroclaw, Poland; ^2^Urology and Urologic Oncology Department, Wroclaw Medical University, Borowska 213, 50-556 Wroclaw, Poland; ^3^Department of Hematology and Bone Marrow Transplantation, School of Medicine in Katowice, Medical University of Silesia, Dabrowskiego 25, 40-032 Katowice, Poland

## Abstract

CD86 molecule is the ligand for both costimulatory (CD28) and coinhibitory (CTLA-4) molecules, and it regulates immune response after allogeneic hematopoietic stem cell transplantation (alloHSCT). Therefore, we postulate that *CD86* gene variations might influence the outcome after alloHSCT. Altogether, 295 adult patients (pts) undergoing related (105 pts) and unrelated (190 pts) donor-matched HSCT were genotyped for the following *CD86* gene polymorphisms: rs1129055, rs9831894, and rs2715267. Moreover, the donors' rs1129055 polymorphism was determined. None of the investigated SNPs alone were associated with aGvHD and rate of relapse. However, we showed that rs2715267 SNP influenced overall survival (OS) after alloHSCT. The 24-month OS for the rs271526GG recipients was worse than that for the recipients possessing T allelle (TT or GT genotypes) (*p* = 0.009). Moreover, analysis of gene-gene interaction between *CD86* and *CTLA-4* showed that having both the A allele for *CD86* rs1129055 and the *CTLA-4* CT60GG genotype in recipients increased the risk of aGvHD about 3.5 times. Interestingly, the donors' rs1129055GG genotype and the recipients' CT60GG genotype also increased the risk of aGvHD about 2.7-fold. We postulate that recipients' *CD86* gene polymorphisms influence the overall survival after alloHSCT and, together with *CTLA-4* polymorphisms, might be considered a risk factor for aGvHD.

## 1. Introduction

Allogeneic hematopoietic stem cell transplantation (alloHSCT) has been established as an effective treatment for patients with hematological malignancies. With the aim of reducing the level of transplantation failure, several studies achieved to elucidate the immunological mechanism involved in this process. Graft-versus-host disease (GvHD) caused by donor-derived T-cells is one of the most common causes of morbidity and mortality after allogeneic HSCT [1]. On the other hand, donor T-cells eliminate malignant residual host T-cells (graft versus leukemia (GvL)) [2]. The balance of alloimmune reaction depending on T-cells is therefore crucial for a successful outcome after allogeneic HSCT. T-cell activation requires two signals. The first signal is antigen specific and required a T-cell receptor (TCR) recognition and binding to MHC/antigen presented by antigen-presenting cells (APC). A second signal (nonspecific) is provided by an interaction between costimulatory molecule CD28 on the T-cell and CD86 and/or CD80 on the APC. This signal leads to clonal T-lymphocyte expansion and differentiation [3]. T-cell activation is downregulated by the binding of CD80 and/or CD86 to cytotoxic T lymphocyte antigen-4 (CTLA-4) [4]. CD86 is constitutively expressed on APC, while CD80 appeared on activated cells. Because protein synthesis level depends on the rate of gene transcription and/or translation, polymorphisms existing in functional sites of genes may affect their expression and/or function. Indeed, an increasing number of studies have demonstrated an important role for polymorphisms in genes encoding molecules from the CD28/CTLA-4/B7 family in organ transplantation [5–10]. For allogeneic HSCT, mostly the associations between *CTLA-4* gene polymorphisms and HSCT outcome were investigated [11–16]. To the best of our knowledge, there are no reports about the role of *CD86* gene polymorphisms in the fate of patients after alloHSCT. Therefore, the aim of our study was to investigate the associations between *CD86* gene variations and alloHSCT outcomes.

## 2. Patients and Methods

### 2.1. Patients

Altogether, 295 adult patients (pts) undergoing related donor- (RD-) matched HSCT (105 pts) and unrelated donor- (URD-) matched HSCT (190 pts) at the Department of Hematology and Bone Marrow Transplantation, Medical University of Silesia, Katowice, between 2006 and 2010 were included in this study. This study was the continuation of the previous work published in immunogenetics [16] and was performed on the same group of HSCT patients.

Primary diagnoses for HSCT patients were as follows: acute myeloid leukemia—147, acute lymphoblastic leukemia—66, chronic myeloid leukemia—18, myelodysplastic syndrome—19, severe aplastic anemia—17, paroxysmal nocturnal hemoglobinuria—11, and other cases—17.

Bone marrow (BM) was the source of hematopoietic stem cells in 124 transplants, and peripheral blood progenitor cells (PBPC) were the source in 163 cases. For 8 recipients, a second transplantation was needed (second transplantation was from RD in 1 case and URD in 7 cases), and for double transplantation, the first source of hematopoietic cells was BM and the second was PBPC.

Myeloablative conditioning (MAC) regimens were based on cyclophosphamide with either busulfan or total body irradiation (TBI) or on melphalan, fludarabine, and alemtuzumab. Reduced-toxicity myeloablative conditioning (RTMAC) was based on treosulfan and fludarabine or cyclophosphamide. Reduced-intensity conditioning (RIC) consisted of fludarabine and busulfan. Antithymocyte globulin (ATG) was used in all recipients of HSCT from unrelated donors. SAA patients received cyclophosphamide and ATG.

The Local Ethics Committee approved this study, and all patients and controls gave their informed consent for the study procedures.

### 2.2. Determination of Polymorphisms

Genomic DNA was isolated from donors' and recipients' frozen whole blood using a QIAamp Blood mini kit (Hilden, Germany). The rs1129055 (named also as CD86+1057G>A or CD86A304T), rs9831894, and rs2715267 (named also as −3479T>G) SNPs were genotyped in recipient samples using the following TaqMan® SNP Genotyping Assays, respectively: C___7504226_10, C_____56422_10, and C__26193522_10 (Applied Biosystems, Foster City, USA). The rs1129055 was genotyped also in donors. Genotyping for SNPs was validated using direct sequencing.

### 2.3. Statistical Analyses

Model coefficients and their 95% confidence intervals (95% CI) were estimated based on *B* = 4900 bootstrap samples. *R* ^2^ coefficient is the fraction of the variation in the response variable explained by the model. Chi-square test, *χ* ^2^_df_, was used to test the null hypothesis that cases and controls have the same distribution of genotype counts. In case of small numbers, distribution of the test statistics was estimated numerically. *Odds ratio* (OR) was computed as the measure of effect size. Departure from Hardy-Weinberg equilibrium (HWE) was tested with the chi-square test. Haplotype frequencies (HFs) among SNPs were estimated with *maximum likelihood* function [17]. The measure for the estimation of pairwise linkage disequilibrium (LD) was squared correlation between two SNPs (*R* ^2^) [17]. For two SNPs, *r* and *R* ^2^ were obtained as $r=D_{ij}/\sqrt{p_{i}q_{j}}$, where *p*_*i*_ and *q*_*j*_ are the population allele frequencies of the *i*th allele on locus *A* and the *j*th allele on locus *B*, *D*_*ij*_ = *x*_*ij*_ − *p*_*i*_ *q*_*j*_, and *x*_*ij*_ is the frequency of the haplotype with alleles *i* and *j* on loci *A* and *B*, respectively. *R* ^2^ = ∑_*i*_^2^∑_*j*_^2^ *D*_*ij*_^2^/*p*_*i*_ *q*_*j*_. Likelihood ratio statistic (LRS) was used to test for differences in haplotype frequencies between cases and controls. LRS = 2(LL_cases_ + LL_controls_ − LL_combined_).

To control type I error in the case of many tests for differences between cases' and controls' SNPs, genotypes adjusted for significance level were estimated. Because of correlation between SNPs, estimation of *α* was performed numerically.

Differences were considered to be statistically significant if the *p* value was < 0.05.

## 3. Results

### 3.1. *CD86* Gene Polymorphism Study

No polymorphism data from donors and recipients demonstrated deviation from Hardy-Weinberg equilibrium.

We observed no linkage disequilibrium between all investigated *CD86* gene polymorphisms (Table 1).

Haplotype analysis showed that the G-C-T haplotype (30.8%) was the most frequently observed in alloHSCT patients. Similar frequency was found for haplotype G-A-G (28%). For A-A-T and G-A-T haplotypes, the frequencies were 16.2% and 14.2%, respectively, and for the other, the frequencies were below 3.5% (Table 2).

### 3.2. The Association between *CD86* Gene Polymorphisms in Recipients and aGvHD

In univariate analysis, we found that none of the investigated *CD86* polymorphisms in recipients were associated with susceptibility to aGvHD (Table 3), although we observed no statistically significant prevalence of carriers of rs1129055A allele among recipients who developed aGvHD (0.50 versus 0.41).

Logistic regression analysis including recipient *CD86* gene variations and other aGvHD prognostic factors [HLA matching, conditioning regimen (myeloablative, RTMAC, or RIC), graft source (BM or PBPC), mode of transplantation (related or unrelated donor), diagnosis (hematological malignances or others), recipient age, donor's sex and age, and polymorphisms in the *CTLA-4* gene] showed no associations between the investigated polymorphisms in the *CD86* gene and susceptibility to aGvHD (data not shown).

### 3.3. The Association between *CD86* Gene Polymorphisms and Relapse

The relapse incidences occurred in 32 patients. Twenty-six of them died due to this complication. None of the recipients' *CD86* gene polymorphisms had impact on the rate of relapse (data not shown).

### 3.4. The Association between *CD86* Gene Polymorphisms and Overall Survival

Median follow-up was 24 months (range: 0.5–67.5). In the present cohort of patients, 198 recipients were alive and 97 died during the observation period. For the living patients, the median OS was 34.23 months (Q1 = 23.36, Q3 = 50.38), while for the dead recipients, the median OS was 5.53 months (Q1 = 2.77, Q3 = 13.48).

The Cox regression analysis showed that the recipients with the rs2715267GG genotype have increased risk of death (HR = 1.93; 95% CI: 1.14–3.08, *p* = 0.009) as compared to the recipients with T allele. The proportion of the living recipients in relation to the rs2715267 genotype is presented in Table 4.

**Figure 1:**
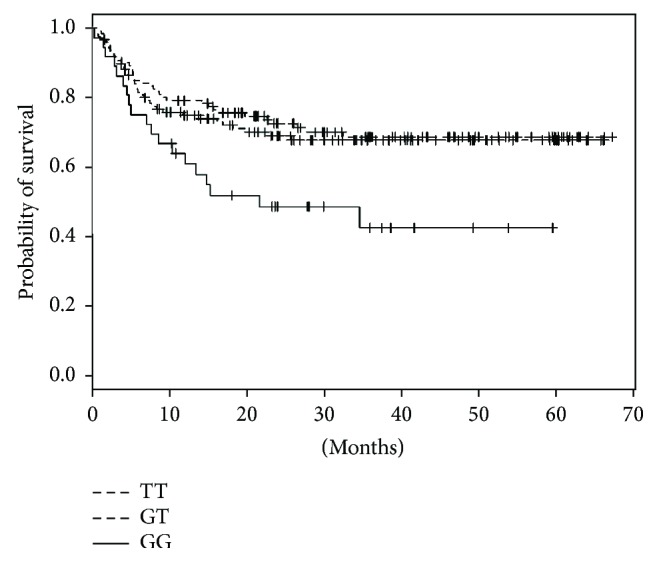
Overall survival in recipients of allogeneic HSCT in relation to rs2715267 polymorphism in the *CD86* gene in recipients.

The presence of the rs2715267GG genotype resulted in worse OS during 24-month observation than did the presence of the TT and GT genotypes, for which the OS was similar (48.4% versus 68.2% and 72.4%, log-rank *p* = 0.009) (Figure 1).

### 3.5. The Association between rs1129055 (CD86+1057G>A) Gene Polymorphism in Donors and aGvHD

In univariate analysis, we found no associations between *CD86* rs1129055 gene polymorphism and risk of aGvHD, although no statistically significant prevalence of GG donors' genotype was observed among recipients who developed aGvHD as compared to patients without aGvHD (54.8% versus 47.8%, Table 3).

### 3.6. An Analysis of Gene-Gene Interaction between *CTLA-4* and *CD86* Polymorphisms and Risk of aGvHD

Based on the results described above and our previous data [16], we analyzed the influence of two factors: factor A (possessing A allele for *CD86* rs1129055 in recipients) and factor B (possessing the GG genotype for CT60 SNP in the *CTLA-4* gene in recipients), on the risk of aGvHD using the Svejgaard and Ryder method [18]. The results of that analysis are presented in Table 5(a). The frequency of carriers of susceptibility alleles for both SNPs (factor A and factor B) was significantly higher in the aGvHD patients as compared to the individuals lacking factors A and B (test [8], Table 5(a)), and possessing both factors A and B increased the risk of disease 3.52-fold (OR 3.52, 95% CI: 1.65–7.53, *p* = 0.001).

Also, based on current and previous results, we used the Svejgaard and Ryder method to assess the influence of a recipient's CT60GG genotype and a donor's rs1129055GG genotype on the risk of aGvHD.

Possession of two susceptible factors: factor A (rs1129055GG genotype in donors) and factor B (CT60GG in recipients), increased the risk of aGvHD 2.73 times (OR 2.73, 95% CI: 1.25–5.95, *p* = 0.01) with respect to those donor-recipient pairs without susceptible factors (Table 5(b)). What is more, the presence of both factors also increased the risk of aGvHD more than 3-fold with respect to the patients possessing CT60A+ allele transplanted from rs1129055GG donors.

## 4. Discussion

Posttransplant T-cell activation plays the key role in alloimmune reactivity that can lead to the recognition of non-self-antigens and cause GvHD or contribute to the elimination of leukemic cells (GVL) and to the prevention of an infection. The balance between alloreactivity and immunotolerance for recipient cells is of great importance. This balance is dependent on the strengths of the costimulatory and coinhibitory signals. In both situations, ligands for costimulatory (CD28) and coinhibitory (CTLA-4) molecules are CD80 and CD86. The role of *CTLA-4* gene polymorphisms in the outcome of HSCT was investigated by us and several other groups of researchers [11, 16, 19–22]. To the best of our knowledge, this is the first report about the association between *CD86* gene polymorphisms and outcome of allogeneic HSCT.

We focused our attention on associations of the three SNPs (rs1129055, rs9831894, and rs2715267) with aGvHD, relapse rate, and overall survival. The selection of SNPs was made on the basis of literature data, in silico analysis, and our previous study.

The results of a *CD86* gene polymorphism study showed no association between the selected SNPs in recipients and donors and aGvHD and rate of relapse. However, we showed that the rs2715267GG genotype resulted in worse OS during 24-month observation than did the presence of the TT and GT genotype.

The rs2715267T>G SNP was previously described by Abdallah et al. [23] as a predisposing factor to systemic sclerosis, and the authors showed that G allele conferred the higher risk of the disease. Moreover, the authors indicated that T allele in this *locus* contains putative binding sites for transcription factor GATA- and TATA-binding protein, which is not the case in the presence of G allele. Also, electrophoretic mobility gel shift assays (EMSA) performed by that group showed that G allele had less binding affinity for nuclear proteins in comparison to T allele.

Moreover, in another immune tolerance-related disease, like asthma [24], the authors made hypothesis that the *CD86* gene, as a part of the vitamin D pathway, is associated with susceptibility to the disease; especially, the rs2715267 SNP was associated with the modest risk of atopy and asthma. Also, for rheumatoid arthritis (RA) [25], this SNP together with −3458A>G *CD40LG* polymorphism was associated with the risk of RA. On the basis of in silico analysis, Lee et al. [25] postulated that predicted binding motifs for *CD86* rs2715267 are SRF, ELF3, TCF3, TCF7, TCF7L2, and ZFP105, but these speculations were not confirmed by any *in vitro* study.

In our work on multiple sclerosis, we found that the presence of G allele (GT or GG genotype) in this SNP was associated with higher risk of this T-cell-dependent disease [26].

On the basis of literature data, we speculate that G allele confers susceptibility to autoimmune disease, which is associated with overstimulation of immune response, which might influence the overall survival of HSCT patients.

The current work includes also data from previous HSCT study on *CTLA-4* gene polymorphisms [16]. On the basis of currently and previously achieved results, we selected two potential pairs for two gene-gene interactions for the analysis of their association with the aGvHD risk. We found that recipients possessing both A allele for rs1129055 in the *CD86* gene and the GG genotype for CT60 SNP in the *CTLA-4* gene had an increased risk of aGvHD about 3.5 times as compared to individuals lacking these variations.

What is more, the *CD86* rs1129055GG genotype in donors and *CTLA-4* CT60GG in recipients also increased the risk of aGvHD about 2.7 times as compared to pairs not carrying susceptible genotypes. Interestingly, possession of *CTLA-4* CT60GG in recipients is of great importance, since comparison between recipients possessing and lacking this factor transplanted from the *CD86* rs1129055GG donor revealed that this factor increased the risk of aGvHD about 3-fold.

The analysis in relation only to *CD86* rs1129055 SNP in donors and recipients showed no statistically significant correlation between the *CD86* rs1129055GG genotype in donors and A allele for *CD86* rs1129055 in recipients (64% versus 49%, *p* = 0.09; Supplementary Material 1).

Surprisingly, opposite genotypes of *CD86* rs1129055 in donors and recipients play a role in the risk of aGvHD.

The *CD86* rs1129055 SNP, previously named CD86+1057G>A or CD86A304T, is the most frequently studied polymorphism in the *CD86* gene. It was discovered in 2000 by Delneste et al. [27] as A to G transition at position 910 on cDNA (starting from ATG codon), causing the exchange of alanine to threonine residue at codon 304. On the basis of genomic sequencing, this SNP was found to be located on 257 bp position downstream of the first nucleotide of exon 8. It is speculated that this transition introduces a potential phosphorylation site in the cytoplasmic region [28].

In the literature, there is a limited number of studies about rs1129055 gene polymorphism and most of them are related to cancer risk. However, the results of the study in various cancers are different. The results for pancreatic cancer [29], for Ewing sarcoma [29], and for osteosarcoma [30] indicated the AA and/or AG genotype as a susceptibility factor, while other studies indicated that individuals carrying the GG genotype were more prone, for example, to colorectal cancer [31]. In the latest meta-analysis, performed on the basis of available literature, the GG genotype was shown to decrease risk of cancer as compared to the AA genotype, especially in Asian population [32].

*CD86* gene variations and rs1129055 were investigated also in the context of chronic obstructive pulmonary disease [33] and brucellosis infections [34], and the AA genotype was indicated as a susceptibility factor for those diseases, while the GG genotype was associated with higher risk of pneumonia-induced sepsis [35]. What is the most interesting for this study is that rs1129055 SNP was found to be associated with the risk of acute rejection after kidney transplantation in Tunisian patients. The AA genotype and possession of A allele were indicated as protective against that complication [8]. Similar results were obtained for liver transplantation. Marín et al. [9] indicated that the AA genotype was not observed in the group of patients with acute rejection episodes. The recent meta-analysis reassuming results of three studies (including the study of de Reuver et al. [7]) confirmed the protective role of AA and A alleles in acute rejection after allografts [10], and *ipso facto*, the GG genotype in immunocompetent cells increased the risk of alloimmunological recognition.

In our study of HSCT, donors' rs1129055GG immunocompetent cells grafted to the recipients also increased the risk of enhanced immune response leading to aGvHD reaction.

On the other hand, the CD86 molecule is involved either in activation of T-cell response or in inhibition of this process. It depends on the balance between CD28 and CTLA-4 binding. Therefore, not only donors' and recipients' CD86 genetic background but also donors' and recipients' *CD28* and *CTLA-4* genetic status might play a role in alloreactivation. As we showed in both gene-gene interaction analyses, recipients' *CTLA-4* CT60GG genotype is an important factor for the risk of aGvHD.

The third polymorphism, rs9831894, investigated by us which was described as the risk factor for Graves' ophthalmopathy in a Taiwanese population [36] was not associated with any complication after allogeneic hematopoietic stem cell transplantation, as well as with overall survival.

The limitation of this study is the heterogenic group of donor-recipient pairs; however, we believe that the implementation of a multivariate logistic regression analysis, which includes other prognostic factors for aGvHD development, allows us to overcome this weakness. Moreover, probably due to the low relapse rate observed in our cohort of patients, we were not able to find any association between donor and recipient *CD86* gene polymorphisms. In addition, due to low amount of material from donors, we were not able to type the same number of SNPs in donors and recipients.

In conclusion, the results of our study suggest that *CD86* gene polymorphisms, especially in recipients, influence the overall survival after HSCT, and also, together with *CTLA-4* gene polymorphism, might be considered a risk factor for aGvHD.

## Figures and Tables

**Table 1:** Linkage disequilibrium between *CD86* SNPs: rs1129055, rs9831894, and rs2715267.

*R* ^2^	rs9831894	rs2715267
rs1129055	0.064	0.016
rs9831894	—	0.142

**Table 2:** The frequencies of haplotypes in recipients for *CD86* gene SNPs: rs1129055, rs9831894, and rs2715267.

Haplotypes (rs1129055, rs9831894, and rs2715267)	Number	Frequency
G-C-T	165.24	0.308
G-A-G	149.96	0.28
A-A-T	87.09	0.162
G-A-T	76.32	0.142
A-C-G	18.92	0.035
A-A-G	16.64	0.031
A-C-T	12.35	0.023
G-C-G	9.49	0.018

**(a):** 

Recipients		aGvHD present		aGvHD absent		OR	95% CI	aGvHD present versus aGvHD absent
		*N*	%	*N*	%			
rs1129055	GG	77	*50.3*	76	*59.4*	1^a^	—	*χ* ^2^_df=2_ = 2.839 *p* = 0.2418
	AG	62	*40.5*	45	*35.2*	1.36	0.83–2.23	
	AA	14	*9.2*	7	*5.5*	1.91	0.75–4.87	
	Σ	153	*100%*	128	*100%*	—		
	Hardy-Weinberg equilibrium							
		*p* = 0.8454		*p* = 0.9199				

rs9831894	AA	57	*39.6*	47	*36.2*	1^a^	—	*χ* ^2^_df=2_ = 0.838 *p* = 0.6578
	AC	63	*43.8*	64	*49.2*	0.81	0.48–1.36	
	CC	24	*16.7*	19	*14.6*	1.04	0.51–2.11	
	Σ	144	*100%*	130	*100%*	—		
	Hardy-Weinberg equilibrium							
		*p* = 0.3801		*p* = 0.854				

rs2715267	TT	69	*44.8*	52	*40*	1^a^	—	*χ* ^2^_df=2_ = 1.378 *p* = 0.5021
	GT	64	*41.6*	63	*48.5*	0.77	0.47–1.26	
	GG	21	*13.6*	15	*11.5*	1.05	0.50–2.21	
	Σ	154	*100%*	130	*100%*	—		
	Hardy-Weinberg equilibrium							
		*p* = 0.3713		*p* = 0.7022				

**(b):** 

Donors		aGvHD present		aGvHD absent		OR	95% CI	aGvHD present versus aGvHD absent
		*N*	%	*N*	%			
rs1129055	GG	80	*54.8*	54	*47.8*	1^a^	— —	*χ* ^2^_df=2_ = 1.839 *p* = 0.3988
	AG	55	*37.7*	46	*40.7*	0.81	0.48–1.36	
	AA	11	*7.5*	13	*11.5*	0.58	0.24–1.36	
	Σ	146	*100%*	113	*100%*	—		
	Hardy-Weinberg equilibrium							
		*p* = 0.6747		*p* = 0.5181				

*χ* ^2^_df≈10_ = 8.521, *p* = 0.5345. ^a^refenence.

**Table 4:** The presentation of overall survival times in relation to the *CD86* rs2715267 genotype in recipients.

Proportion of survivors	95%	90%	85%	75%
rs2715267	Time (months)			
TT	2	3.83	5.53	18.38
TG	2.33	3.73	5.47	11.77
GG	1.43	2.3	3.5	5.07

*χ* ^2^_df=3_ = 17.81; *p* = 0.0005; *R* ^2^ = 0.063.

**Table:** (a) Factor A (carriers of A alleles for *CD86* rs1129055 in recipients) and factor B (possessing the CT60GG (rs3087243 GG) genotype for the *CTLA-4* gene in recipients)

	aGvHD present		aGvHD absent		
A+B+	35		13		
A−B+	32		25		
A+B−	29		36		
A−B−	39		51		

Test	OR	*p*	95% CI	Comparison	Individual association
(1) A	1.58	0.07	0.97–2.58		
(2) B	**2.25**	**0.002**	**1.35–3.75**		
(3) ++ versus −+	2.10	0.08	0.92–4.80	A in B-positive	A association
(4) +− versus −−	1.05	0.87	0.55–2.00	A in B-negative	
(5) ++ versus +−	**3.34**	**0.003**	**1.49–7.46**	B in A-positive	B association
(6) −+ versus −−	1.67	0.13	0.86–3.27	B in A-negative	
(7) +− versus −+	0.63	0.21	0.31–1.29	Differences between A and B association	
(8) ++ versus −−	**3.52**	**0.001**	**1.65–7.35**	Combined association	

**Table:** (b) Factor A (carriers for the *CD86* rs1129055GG genotype in donors) and factor B (possessing the CT60GG (rs3087243GG) genotype for the *CTLA-4* gene in recipients)

	aGvHD present		aGvHD absent		
A+B+	36		14		
A+B−	32		41		
A−B+	26		21		
A−B−	33		35		

Test	OR	*p*	95% CI	Comparison	Individual association
(1) A	1.l7	0.54	0.70–1.95		
(2) B	**2.07**	**0.007**	**1.22–3.52**		
(3) ++ versus −+	2.08	0.09	0.89–4.83	A in B-positive	A association
(4) +− versus −−	0.83	0.58	0.43–1.61	A in B-negative	
(5) ++ versus +−	**3.29**	**0.002**	**1.52–7.12**	B in A-positive	B association
(6) −+ versus −−	1.31	0.47	0.62–2.77	B in A-negative	
(7) +− versus −+	0.63	0.22	0.30–1.32	Differences between A and B association	
(8) ++ versus −−	**2.73**	**0.02**	**1.25–5.95**	Combined association	

## Supplementary Materials

Supplementary Materials Supplementary Material 1: an analysis of the associations between two genetic factors and risk of aGvHD using the Svejgaard and Ryder method [19]: factor A: CD86 rs1129055GG genotype in donors; factor B: possessing A allele for CD86 rs1129055 in recipients (AG or AA genotype).
