# Supplementary material for: The Influence of Genetic Variations in the CD86 Gene on the Outcome after Allogeneic Hematopoietic Stem Cell Transplantation
Source: J Immunol Res. 2018 Feb 7;2018:3826989. doi: 10.1155/2018/3826989 (PMC5821961; doi:10.1155/2018/3826989)
Supplement: Supplementary Materials — Supplementary Material 1: an analysis of the associations between two genetic factors and risk of aGvHD using the Svejgaard and Ryder method [19]: factor A: CD86 rs1129055GG genotype in donors; factor B: possessing A allele for CD86 rs1129055 in recipients (AG or AA genotype). [file 3826989.f1.docx]

Supplementary material 1:

An analysis of the associations between two genetic factors and risk of aGvHD using Svejgaard and Ryder method [19]- Factor A: *CD86* rs1129055GG genotype in donors; Factor B: possessing of A allele for *CD86* rs1129055 in recipients (AG or AA genotype)

|  | Factor A: ***CD86* rs1129055 GG genotype in donors**  Factor B: **possessing of A allele for *CD86* rs1129055 in recipients (AG or AA genotype)** | | | | |
| --- | --- | --- | --- | --- | --- |
|  | aGvHD present | | aGvHD absent |  | |
| A+B+ | 36 | | 20 |  | |
| A+B- | 38 | | 36 |  | |
| A-B+ | 34 | | 25 |  | |
| A-B- | 31 | | 32 |  | |
|  | | | | | |
| Test | OR | *p* | 95%CI | Comparison | Individual association |
| [1] A | 1.15 | 0.56 | 0.71-1.91 |  |  |
| [2] B | 1.23 | 0.40 | 0.75-2.00 |  |  |
| [3] ++ *vs* -+ | 1.32 | 0.47 | 0.62-2.80 | A in B-positive | A association |
| [4] +- vs -- | 1.09 | 0.80 | 0.56-2.13 | A in B-negative |  |
| [5] ++ vs +- | 1.71 | 0.14 | 0.84-3.48 | B in A-positive | B association |
| [6] -+ vs -- | 1.40 | 0.35 | 0.69-2.87 | B in A-negative |  |
| [7] +- vs -+ | 0.78 | 0.47 | 0.39-1.55 | Differences between A and B association |  |
| [8] ++ vs -- | 1.86 | 0.10 | 0.89-3.88 | Combined association |  |
|  |  |  |  |  |  |
